# Mechanical instability induces osteoclast differentiation independent of the presence of a fibrous tissue interface and osteocyte apoptosis in a rat model for aseptic loosening

**DOI:** 10.1080/17453674.2019.1695351

**Published:** 2019-11-25

**Authors:** Rune Vinther Madsen, Denis Nam, Jörg Schilcher, Aleksey Dvorzhinskiy, James P Sutherland, F Mathias Bostrom, Anna Fahlgren

**Affiliations:** a Hospital for Special Surgery, New York, USA;; b Rush University Medical Center, Chicago, USA;; c Department of Clinical and Experimental Medicine, Linköping University, Linköping, Sweden;; d Department of Orthopaedic Surgery, Zealand University Hospital, Køge, Denmark;; e Department of Orthopedic Surgery, University Hospital Linköping, Sweden

## Abstract

Background and purpose — Insufficient initial fixation or early micromotion of an implant is associated with a thin layer of fibrous tissue at the peri-implant interface. It is unknown if bone loss is induced by the fibrous tissue interface acting as an active biological membrane, or as a membrane that will produce supraphysiologic fluid flow conditions during gait, which activates the mechanosensitive osteocytes to mediate osteoclast differentiation. We investigated whether mechanically induced osteolysis is dependent on the fibrous tissue interface as a biologically active scaffold, or if it merely acts as a conduit for fluid flow, affecting the mechanosensitive osteocytes in the peri-prosthetic bone.

Methods — Using a rat model of mechanically instability-induced aseptic loosening, we assessed whether the induction of osteoclast differentiation was dependent on the presence of a peri-implant fibrous interface. We analyzed the amount of osteoclast differentiation, osteocyte apoptosis, pro-resorptive cytokine expression and bone loss using immunohistochemistry, mRNA expression and micro-CT.

Results — Osteoclast differentiation and bone loss were induced by mechanical instability but were not affected by the presence of the fibrous tissue membrane or associated with osteocyte apoptosis. There was no increased mRNA expression of any of the cytokines in the fibrous tissue membrane compared with the peri-implant bone.

Interpretation — Our data show that the fibrous tissue membrane in the interface plays a minor role in inducing bone loss. This indicates that the peri-implant bone adjacent to loose bone implants might play an important role for osteoclast differentiation.

Implant micromotion during the first 6 months after implantation is associated with an increased risk of later prosthetic loosening (Pijls et al. 2012, Streit et al. 2016). The synovial-like fibrous tissue membrane in the peri-prosthetic interface has been made responsible for this loosening process through several mechanisms: a reservoir for wear debris particles; accumulation of inflammatory cells including release of pro-osteoclastic factors (Ingham and Fisher 2005); a tissue layer causing mechanical instability and increased micromotion.

Because the amount of wear debris particles does not correlate with bone loss severity at the peri-prosthetic interface, the fibrous tissue membrane may serve as a conduit transmitting pressurized fluid flow with or without wear-debris particles to the bone–implant interface (Alidousti et al. 2011) leading to osteoclastogenesis (Cyndari et al. 2017, Holding et al. 2006, Mandelin et al. 2005).

Resorption typically starts at the implant (or cement)–bone interlock and progresses from there into the surrounding bone (Goodheart et al. 2017). Because this interlock and specifically osteolytic zones are exposed to supraphysiologic levels of fluid shear stress (Mann and Miller 2014), these areas might be responsible for the initiation of bone loss. This pressurized fluid flow already induces osteoclast differentiation and bone loss within five days (Skripitz and Aspenberg 2000, Fahlgren et al. 2010, Aspenberg et al. 2011, Nilsson et al. 2012).

However, it is still unknown whether the fibrous tissue initiates the osteolytic response in the peri-implant bone or if the signals come from elsewhere such as osteocyte apoptosis (Matsumoto et al. 2013, Kennedy et al. 2014).

We hypothesized that osteolysis depends on osteoclast differentiation induced through the peri-implant fibrous tissue interface. To test this hypothesis and whether mechanically induced osteoclast differentiation is associated with osteocyte apoptosis, we used a rat model for instability-induced aseptic loosening.

## Material and methods

### Animal model and surgical procedure

**Figure 1.**
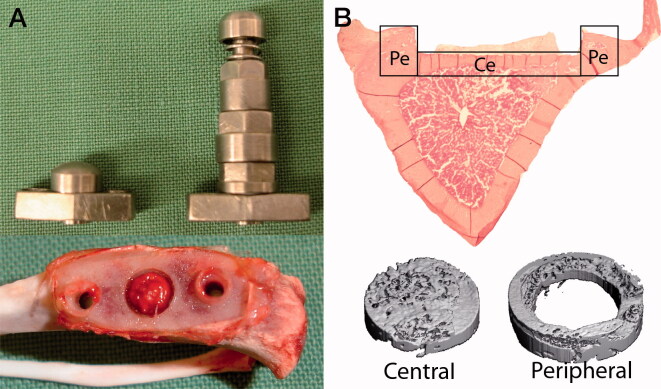
Overview of implants for instability-induced osteolysis and the studied rat tibia. A. The central screw (S) and the pressure piston (P) that are placed in the titanium plate and implanted in the proximal tibia. Retrieved proximal tibia demonstrating the fibrous tissue that forms beneath the piston. B. Histological section with the central (Ce) cortical bone underneath the pressure piston and the peripheral (Pe) cortical bone at the periphery. MicroCT images illustrating the central and peripheral bone volumes.

We used 45 Sprague-Dawley male rats (mean weight 404 g, SD 24 g) in our previously described model (Skripitz and Aspenberg 2000, Fahlgren et al. 2010) (Figure 1).

**Figure 2.**
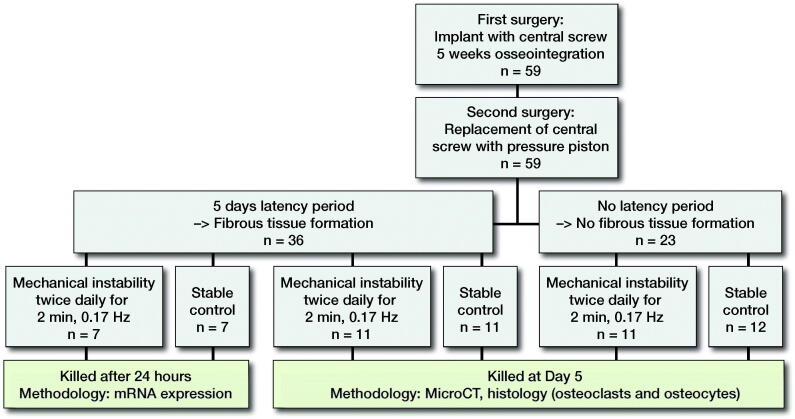
Study design. The role of fibrous tissue for mechanically induced osteoclast differentiation and osteolysis (left arm), and the association between osteocyte apoptosis and osteoclast differentiation (right arm). Micro-CT performed at day 5.

General anesthesia was induced by intra-peritoneal injection of a mixture of ketamine (80–90 mg/kg) and xylazine (5 mg/kg), and prolonged by the administration of inhaled isoflurane via a nose cone. A depression in the tibial cortex was milled out to correspond to the pressure area under the piston. After the depression was prepared, an implant with the central screw (S) was secured to the predominantly cortical bone. The central plug was replaced by the pressure piston (P) (Figure 1A) during a second surgery, after 5 weeks of osseointegration. Buprenorphine at 0.01–0.05 mg/kg was given twice daily for the first 2 postoperative days.

### Presence or absence of fibrous tissue (Figure 2)

5 weeks after the implant with the central plug was inserted, we divided all animals into 2 groups. In the first group no piston was inserted for the first 5 days before we initiated mechanical instability. During these 5 days a fibrous tissue layer forms in the space between the piston and the bone surface (Figure 1A) (Skripitz and Aspenberg 2000, Fahlgren et al. 2010). In the second group, mechanical instability was initiated the day after the piston was placed, thus preventing fibrous tissue formation. Each of the 2 groups was further divided into a group with mechanical instability and one unloaded group (controls). In the groups of mechanical instability, the piston was loaded with 0.6 MPa twice daily for 2 minutes at a frequency of 0.17 Hz. This mode of loading is associated with similar levels of osteoclast differentiation in mechanical instability and wear debris particles (Nilsson et al. 2012, Amirhosseini et al. 2018). Animals were killed 5 days after the first loading episode and at the same time in surgically treated non-loaded controls.

#### MicroCT

We used micro-computed tomography (µCT) using a Scanco uCT 35 (Scanco Medical, Bruttisellen, Switzerland) to scan the experimental area under the piston with a resolution of 15 µm. The tibiae were placed in a saline-filled tube and scanned at 55 kV and 145 mA. The Scanco µCT software (HP, DECwindows Motif 1.6) was used for 3D reconstruction and viewing of the images. There were 2 regions of interest for each specimen: (1) a central zone (Ce), comprising a 3 mm area down to 1.2 mm depth underneath the piston; (2) a peripheral zone (Pe), 0.75 mm from the periphery of the central zone down to 1.2 mm deep (Figure 1B). The bone volume (BV) was measured in each zone.

### Histological analysis and immunolocalization of osteoclasts and osteocytes

Proximal tibiae were decalcified, embedded in paraffin, and cut in transverse sections of 7 µm thickness (Nilsson et al. 2012). Each specimen was stained for rabbit-anti rat Cathepsin K (1:600) (Zenger et al. 2007) to identify the number of osteoclasts, and Cleaved Caspase-3 (#9661, Cell Signaling Technologies, Carpinteria, CA, USA) to identify apoptotic osteocytes. Detection of Cathepsin K was done as previously described (Nilsson et al. 2012). For Cleaved Caspase-3, sections were treated for 30 minutes with a methanol-NaOH solution for antigen retrieval (DeCal, Biogenex, San Ramon, CA, USA), then blocked for another 30 minutes with serum free Protein Block. Sections were incubated overnight at 4 °C with primary antibodies and detected by a biotinylated antibody (1:600) for 40 minutes. Vectastain Elite ABC kit (Vector Laboratories, Burlingame, CA, USA) was applied for 30 minutes followed by 5 minutes of 3,3´diaminobenzidine (DAB) (Sigma, St Louis, MO, USA). The sections were then counterstained, dehydrated, and mounted.

The number of osteoclasts was defined as multinucleated Cathepsin K positive cells within a distance of 0.1 mm from the bone surface and counted at an objective of x10 magnification. The number of stained and unstained osteocytes was counted using x40 magnification objective. Cleaved Caspase-3 stained osteocytes were counted as apoptotic osteocytes and empty lacunae were counted as osteocyte necrosis. Apoptotic and necrotic osteocytes are shown as a percentage of total osteocytes. Osteoclast numbers and osteocyte apoptosis and necrosis were evaluated at both the central and peripheral location. A blinded investigator analyzed 1 representative section from 2 levels of the cortical bone with a distance of 450 µm.

### RNA isolation and gene expression analysis of the fibrous tissue and the peri-prosthetic bone

Using the same animal model, 14 male Sprague-Dawley rats (mean weight 380 g, SD 16 g), were subdivided into mechanical instability and non-loaded controls. Animals in both groups were killed 24 hours after the first loading episode. For RNA isolation, custom-made trephines with diameters of 2.0 mm and 4.5 mm respectively were used to harvest bone from the central and peripheral locations in the loaded areas (Figure 1B). The bone marrow was carefully removed from the bone specimens. No periosteum was present as this was trimmed away during surgery. The fibrous tissue and the central and peripheral bone sections were snap-frozen separately in liquid nitrogen. Extraction of total RNA was performed using the TRIspin method described in detail (Nilsson et al. 2012). Primers for IL-6 (NM_012589), HIF-1α (NM_024359), SOST (NM_030584), RANKL (NM_057149), and OPG (NM_012870) were purchased from Qiagen (Germantown, MD, USA). Amplification was performed in 20 µL reactions using SyberGreen MasterMix (Qiagen). Each sample was analyzed in duplicate. Real-time (q-) PCR reactions were conducted using a standard curve methodology to quantify the specific gene targets of interest and normalized to 18SrRNA. The standard curve was made with rat spleen (Zyagen, San Diego, CA, USA) for IL-6 and HIF-1α or a rat embryo (Zyagen, San Diego, CA, USA) for SOST, RANKL, and OPG.

#### Statistics

Data were collected in a blinded fashion. Primary outcome was osteoclast number. Differences in bone volume, osteoclast number, and osteocyte variables were analyzed using a 2-way ANOVA analysis, with the presence or absence of loading and fibrous tissue as variables. The Mann–Whitney U-test was used to analyze gene expression, which was distributed in a non-parametric manner. Pearson’s correlation was used to determine the relationship between osteocyte apoptosis and osteoclast number.

The expression pattern of the cytokines in the loaded fibrous tissue was within the same range as the unloaded fibrous tissue. Due to technical difficulties during tissue preparation, only 2 out of 6 of the control fibrous tissue samples were analyzed for mRNA expression. Therefore, the values are presented as absolute values and no statistics are presented.

### Ethics, funding, and potential conflicts of interest

All experiments for the first part of the study, involving microCT, histological analysis, and immunolocalization, were carried out with IACUC approval at the Hospital for Special Surgery (Approval date: September 1, 2011, Dnr 09-11-10R).

All experiments for the second part of the study, involving RNA isolation and gene expression analysis, were carried out within the context of institutional guidelines for care and treatment of experimental animals after approval from the Linköping Ethical Committee on animal research (Approval date: November 13, 2012; Dnr 99-12).

The project was supported by the Swedish Research Council; Grant numbers: 521-2013-2593, 2016-01822, 2016-06097; Sweden’s Innovation Agency Grant number: 2012-04409 and the National Institutes of Health Grants R01-AR056802, R01-AG028664, and P30-AR046121.

The authors declare that they have no competing interests regarding this study.

## Results

Presence of fibrous tissue interface had no effect on instability-induced osteoclast numbers and bone loss

**Figure 3.**
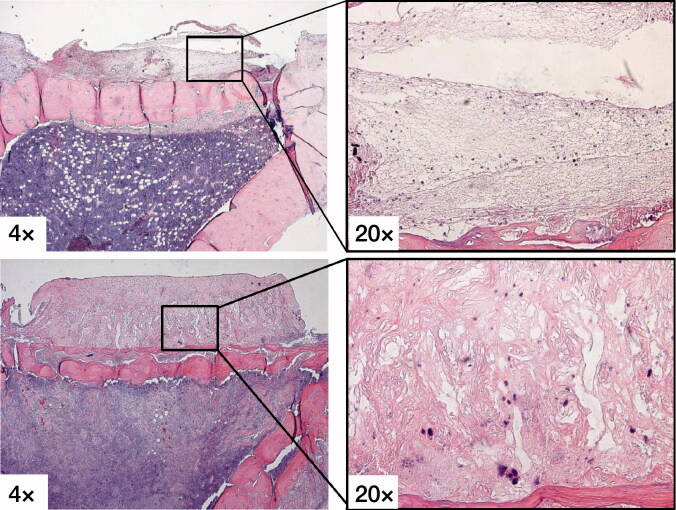
H&E stains showing the fibrous tissue membrane in the immediate loading group (upper row) with a fibrin clot with loose connective tissue after 5 days of loading, while the 5 days latency group (lower row) had dense richly vascularized fibrous tissue.

The fibrous tissue membrane in the immediate loading group showed a fibrin clot with loose connective tissue after 5 days of loading, while the 5 days latency groups had dense richly vascularized fibrous tissue (Figure 3).

**Figure 4.**
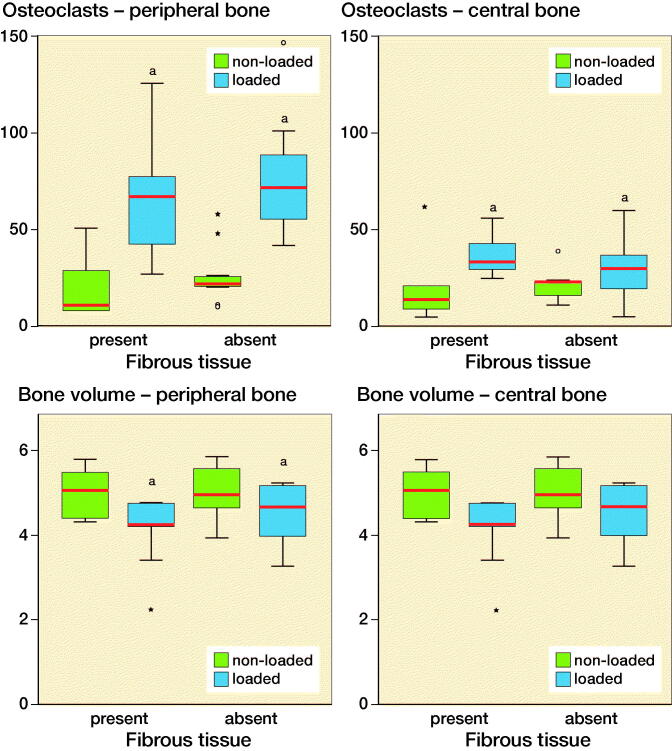
Number of osteoclasts and bone volume in the peripheral and central cortical bone. The results are subdivided based on the presence of the fibrous tissue. Blue-colored boxes indicate loaded specimens, while green-colored boxes refer to non-loaded specimens. **^a^**indicates p < 0.05. Red lines are median, boxes IQR, and whiskers ±1.5 x IQR.

Mechanical instability of the implant increased the number of Cathepsin K positive osteoclast (p < 0.05) after 5 days both underneath the implant (central area) and in the periphery (peripheral area). In the peripheral bone area mechanical instability decreased bone volume. The number of osteoclasts and bone volume did not differ whether fibrous tissue was present or absent under the bone implant (Figure 4).

Osteocyte apoptosis was suppressed by mechanical instability and did not correlate with the numbers of Cathepsin K positive osteoclasts

To determine whether osteocyte apoptosis or necrosis was associated with osteoclast differentiation during instability-induced osteolysis, we counted the amount of Caspase-3 positive osteocytes and empty osteocyte lacunae in relation to the total number of osteocytes. Osteocyte apoptosis was suppressed by mechanical instability underneath the piston (central location) compared with non-loaded controls but remained unaffected in the peripheral area.

Osteocyte apoptosis and necrosis was more frequent in animals with the fibrous tissue present compared with animals without (Table 1).

**Table 1.** Percentage (SD) of osteocyte apoptosis and necrosis in the peri-prosthetic bone 5 days after the first loading session and in non-loaded controls

	Loaded Fibrous tissue		Non-loaded Fibrous tissue	
Factor/location	present	absent	present	absent
Osteocyte apoptosis (%):				
Peripheral	6.9 (4.7)	4.0 (4.1)	6.9 (7.0)	2.7 (1.6)
Central^a^**^, b, c^**	3.0 (0.7)	4.2 (2.7)	4.3 (3.1)	7.9 (4.5)
Osteocyte necrosis (%):				
Peripheral^b^	9.8 (4.8)	6.5 (2.8)	5.5 (3.3)	5.7 (2.2)
Central^b^	14.7 (6.0)	12.6 (8.1)	17.8 (6.7)	14.2 (4.1)

a Effect of loading: p = 0.04

b Effect of fibrous tissue: p < 0.02

c Effect of loading and fibrous tissue: p = 0.07.

The percentage of apoptotic or necrotic osteocytes did not correlate with the number of Cathepsin K positive osteoclasts.

The fibrous tissue had similar mRNA expression of inflammatory factors compared with the bone tissue

To determine whether instability increased gene expression related to osteoblast–osteoclast differentiation, we detected the pro-osteoclastic factors IL-6, HIF-1α and VEGF and SOST, RANKL and OPG. The total expression due to exposure of mechanical instability was compared between bone and fibrous tissue.

Peri-prosthetic bone exposed to mechanical instability had a 5-times higher mRNA expression of IL-6 at the central location (p < 0.05), and 7-times increased expression at the peripheral location (p = 0.07). HIF-1α mRNA expression was increased 2-times (p < 0.05) in the peripheral bone of the mechanical instability group but remained unaffected in the central location. Mechanical instability did not affect HIF-1α in the fibrous tissue. SOST appeared suppressed 13-times (p = 0.07) in the central bone when compared with unloaded controls, while unaffected in the periphery. The fibrous tissue had a 60–600-fold lower expression of SOST after being exposed to mechanical instability compared with the bone tissue. RANKL and OPG ratio was not changed by loading at 24 hours (Table 2).

**Table 2.** Median of mRNA expression in the fibrous tissue and peri-prosthetic bone 24 hours after the first loading session

	Central bone Loaded		Peripheral bone Loaded		Fibrous tissue Loaded	
	yes	no	yes	no	yes	no
Gene	(n = 7)	(n = 5)	(n = 6)	(n = 6)	(n = 7)	(n = 2)
IL-6	0.56 **^a^**	0.11	0.96 **^b^**	0.13	0.68	(0.09, 2.42)
HIF-1	4.25	1.22	2.95 **^a^**	1.67	2.38	(0.72, 1.69)
SOST	0.62 **^b^**	7.88	6.00	9.60	0.01	(1.88, 0.08)
VEGF	0.38	0.12	0.21	0.16	0.44	(0.20, 0.11)
RANKL	5.12	1.84	5.90	2.02	0.34	(0.29, 2.35)
OPG	1.11	0.33	0.52	0.54	0.08	(0.06, 0.14)

a p < 0.05,

b p < 0.07 when loaded samples were compared with non-loaded controls

## Discussion

We hypothesized that the peri-implant fibrous tissue membrane would be responsive to mechanical instability and excrete osteoclast-stimulating factors that would induce bone loss. Contradictory to this hypothesis, we found osteoclast differentiation and bone loss unaffected by the presence or absence of the fibrous tissue membrane. We also found that osteoclast differentiation and bone loss was independent of osteocyte apoptosis and necrosis. Furthermore, we found similar levels of pro-inflammatory gene expression in the fibrous tissue membrane and the underlying bone after mechanical instability.

In a previous study, with the same design as in the current study, we tested the importance of the soft tissue membrane by tearing it apart twice daily for 5 days with rotating movements of a piston with a sharp undersurface. In that study, no pressurized fluid flow was created (Fahlgren et al. 2010). After 5 days the soft tissue underneath the piston was richly vascularized with an abundance of inflammatory cells and endothelial cells. None of the specimens with traumatized tissue showed bone resorption. However, in a control group, where mechanical instability was induced in the same way as in this study, we found peri-implant bone loss. In this peri-implant bone, increased expression of HIF-1α and IL-6 suggests an inflammatory pathway crucial for osteoclast differentiation and bone loss. Low oxygen tension and increased expression of HIF-1α is generally present at sites with increased bone resorption. Hypoxia induces the formation of blood vessel and osteoclast differentiation (Dandajena et al. 2012). An increased expression of IL-6 is likely associated with increased amount of osteoclast differentiation in instability-induced osteolysis (Nilsson et al. 2012, Amirhosseini et al. 2018). IL-6 plays a role in osteoclast and osteoblast differentiation and it has been found to be elevated in the synovial fluid of patients undergoing implant revision surgery (Wang et al. 2010). Variation of the IL-6 gene has also been shown to increase the likelihood of aseptic loosening following hip arthroplasty (Gordon et al. 2010). The current study does not allow us to differentiate properties of the fibrous tissue membrane between early failed osseointegration and late loosening. However, it demonstrates that pro-inflammatory factors in the bone tissue underneath the piston are at similar levels to those in the fibrous tissue membrane.

Osteoblasts and osteocytes respond differently to fluid flow profiles, thus resulting in differences in gene expression and phenotypes (Ponik et al. 2007). In an animal model for fatigue-induced bone remodeling it was shown that neighboring cells to the apoptotic osteocytes in rat ulna trigger osteoclast differentiation and bone remodeling (Kennedy et al. 2012). However, in the current study, we were not able to demonstrate an association between osteocyte apoptosis and the number of differentiated osteoclasts. This suggests that other factors released by osteocytes or osteoblasts might affect osteoclast differentiation. The area of bone destruction is often localized near the arthroplasty implant and may not be representative of the patient’s overall bone quality. Even in the current study, we found local differences due to loading. It was established that the osteocytes beneath the loaded implant had less apoptosis than in the peripheral location. Since the bone below the piston is in effect a transversely loaded thin plate, it is likely that it is in a state of high stress and strain due to bending. This could initiate an anabolic mechanism, counteracting the osteolytic mechanism (Thompson et al. 2012). At this location, there was a tendency of almost a 13-fold mRNA down-regulation of SOST, which is secreted by osteocytes and inhibits osteoblast activity. Similarly, the obligate role of a down-regulation of SOST in osteogenesis was shown in transgenic mice with a persistently high level of SOST during loading, where load-induced bone formation was reduced by 70–85% (Tu et al. 2012). Further, enhanced release of nitric oxide by osteocytes during loading partly regulates osteocyte apoptosis in the neighboring cells by Bcl-2 and Caspase 3 (Tan et al. 2008). This is a suggested mechanism to inhibit bone degradation due to unloading.

The current study examined gene expression, knowing that gene and protein expression do not always correlate due to post-transcriptional and post-translational changes. However, it was outside this study’s capacity to elaborate on these differences. Due to technical difficulties during tissue preparation, we were only able to analyze 2 out of 6 of the control fibrous tissue samples for mRNA expression. Still, the results from these specimens were in the same range as the loaded samples. Therefore, we allowed ourselves to exclude an increased expression of mRNA in the genes of interest. The expression in the fibrous tissue membrane was not higher than in the bone tissue, neither was the expression high enough to have an impact on the number of osteoclasts. Another limitation is that the fibrous tissue used in the current animal model might not accurately represent the chronic granulation tissue seen around the loose prosthesis in human. However, it might represent soft tissue seen around implants in an earlier stage. Although the current study separated the fibrous tissue and the bone tissue samples for qPCR, analyses still include a mixture of cell types. Osteoblasts and osteocytes respond differently to different fluid flow profiles, thus resulting in differences in gene expression and phenotypes (Ponik et al. 2007).

In conclusion, it appears that the main role of the fibrous tissue is to provide a conduit for fluid flow in instability-induced osteolysis, rather than acting as a biologically active scaffold itself. Osteoclast differentiation and bone loss were induced by mechanical instability but not affected by the presence of the fibrous tissue membrane or associated with osteocyte apoptosis. The ability of the peri-prosthetic bone to induce osteolysis even in the absence of a fibrous tissue membrane may be important. We believe that the current study can provide important information to understand the role of fibrous tissue and the peri-prosthetic bone in the early process of micromotion that later will develop into prosthetic loosening.
